# TREM2-Transduced Myeloid Precursors Mediate Nervous Tissue Debris Clearance and Facilitate Recovery in an Animal Model of Multiple Sclerosis

**DOI:** 10.1371/journal.pmed.0040124

**Published:** 2007-04-10

**Authors:** Kazuya Takahashi, Marco Prinz, Massimiliano Stagi, Olga Chechneva, Harald Neumann

**Affiliations:** 1 Neural Regeneration Unit, Institute of Reconstructive Neurobiology, University of Bonn Life & Brain Center and Hertie-Foundation, Bonn, Germany; 2 Neuroimmunology Unit, European Neuroscience Institute Göttingen, Göttingen, Germany; 3 Department of Neuropathology, University Hospital Göttingen, Göttingen, Germany; 4 Institute of Multiple Sclerosis Research, University of Göttingen and Hertie-Foundation, Germany; Imperial College London, United Kingdom

## Abstract

**Background:**

In multiple sclerosis, inflammation can successfully be prevented, while promoting repair is still a major challenge. Microglial cells, the resident phagocytes of the central nervous system (CNS), are hematopoietic-derived myeloid cells and express the triggering receptor expressed on myeloid cells 2 (TREM2), an innate immune receptor. Myeloid cells are an accessible source for ex vivo gene therapy. We investigated whether myeloid precursor cells genetically modified to express TREM2 affect the disease course of experimental autoimmune encephalomyelitis (EAE), an animal model of multiple sclerosis.

**Methods and Findings:**

EAE was induced in mice by immunization with a myelin autoantigen. Intravenous application of TREM2-transduced bone marrow–derived myeloid precursor cells at the EAE peak led to an amelioration of clinical symptoms, reduction in axonal damage, and prevention of further demyelination. TREM2-transduced myeloid cells applied intravenously migrated into the inflammatory spinal cord lesions of EAE-diseased mice, showed increased lysosomal and phagocytic activity, cleared degenerated myelin, and created an anti-inflammatory cytokine milieu within the CNS.

**Conclusions:**

Intravenously applied bone marrow–derived and TREM2-tranduced myeloid precursor cells limit tissue destruction and facilitate repair within the murine CNS by clearance of cellular debris during EAE. TREM2 is a new attractive target for promotion of repair and resolution of inflammation in multiple sclerosis and other neuroinflammatory diseases.

## Abstract 2

Myeloid precursors genetically modified to express TREM2 supported recovery in animals with experimental autoimmune encephalomyelitis by phagocytosis and removal of apoptotic cellular debris.

## Editors' Summary

**Background.:**

Multiple sclerosis (MS) is a progressive disease of the central nervous system (CNS; the brain and spinal cord) in which communications within the brain are disrupted. In healthy individuals, the brain controls the body by sending electrical messages along nerve fibers. Like electrical wires, these fibers have to be insulated to allow the efficient transfer of electrical impulses. This insulation is provided by myelin, a fatty tissue that surrounds the nerve fibers. In patients with MS, patchy myelin loss, inflammation, and scarring (sclerosis) disrupt nerve transmissions, causing weakness or paralysis of the limbs, balance and coordination problems, and numerous other symptoms. The damage to the myelin (which increases over time) is caused by the patient's immune system, but what triggers this abnormal behavior is unclear. There is no cure for MS, but drugs that modulate the immune system (for example, interferons and steroids) can slow its progression.

**Why Was This Study Done?:**

Any treatment for MS needs to limit tissue destruction by the immune system and to promote the repair of the damaged myelin and nerve fibers. Although researchers have identified some agents that promote nerve repair, it is difficult to get them into the CNS, which is protected by an impermeable barrier. However, some cell types can migrate into the CNS so it might be possible to adapt them as delivery vehicles for therapeutic agents. In this study, the researchers investigated whether bone marrow–derived myeloid precursor cells (a source of several immune system cell types) might be useful for the treatment of MS, both as delivery vehicles and as a source of cells that can resolve inflammation and clear cellular debris from sites of myelin destruction; this clearance has to occur before nerves and myelin can be repaired. Myeloid precursor cells, the researchers reasoned, might satisfy both these requirements because they naturally migrate into the CNS where some turn into microglia, immune system cells that engulf and digest cellular debris.

**What Did the Researchers Do and Find?:**

The researchers isolated myeloid precursor cells from mouse bone marrow and induced the expression of triggering receptor expressed on myeloid cells-2 (TREM2, a protein made by microglia) in them using a viral vector. They injected these TREM2-expressing cells into the veins of mice with experimental autoimmune encephalomyelitis (EAE, an animal model of MS) and examined their migration into the spinal cord, their effect on EAE symptoms, and their effect on cell debris clearance and inflammatory responses in spinal cord lesions. Neither TREM2-expressing nor control myeloid precursor cells migrated into the spinal cord when injected into healthy mice or into animals just beginning to show the symptoms of EAE. However, both cell types migrated into the spinal cord when injected into animals when EAE symptoms were at their peak. The injection of TREM2-expressing myeloid precursor cells (but not control myeloid precursor cells) at this time reduced EAE symptoms and nerve damage, and halted myelin loss. It also increased the clearance of cell debris and myelin fragments and created an anti-inflammatory environment in the spinal cord lesions.

**What Do These Findings Mean?:**

These findings indicate that the intravenous injection of bone marrow–derived myeloid precursor cells engineered to express TREM2 can reduce clinical symptoms and induce recovery in mice with EAE. Whether the TREM2-expressing myeloid precursor cells act locally in the CNS or indirectly via other immune system cells is not clear yet. In addition, the findings need to be replicated in more animals before this approach is tested in people. Nevertheless, the intravenous injection of cells of this type could provide a two-pronged approach to the treatment of MS (and other neuroinflammatory diseases). First, bone marrow–derived myeloid precursor cells that have been engineered to express TREM2 could help to treat MS by clearing tissue debris and reducing inflammation in spinal cord lesions. Second, the same cells could be used to deliver agents to the lesions that promote nerve repair. Importantly, these results suggest that TREM2-expressing myeloid precursor cells might have the potential to treat MS after the onset of clinical symptoms, an important consideration for a chronic disease that often indicates relapses by new clinical symptoms.

**Additional Information.:**

Please access these Web sites via the online version of this summary at http://dx.doi.org/10.1371/journal.pmed.0040124.

- US National Institute of Neurological Disorders and Stroke patient information on multiple sclerosis (in English and Spanish)
- UK National Health Service health encyclopedia information on multiple sclerosis
- MedlinePlus encyclopedia pages on multiple sclerosis
- The US National MS Society information on multiple sclerosis for patients and professionals (in English and Spanish)
- UK MS Society  information on multiple sclerosis for patients and professionals
- Multiple Sclerosis International Federation information on multiple sclerosis for patients, caregivers, and professionals (in English, Arabic, German, Spanish, French, Italian and Russian)

## Introduction

Hematopoietic-derived myeloid cells physiologically migrate into the central nervous system (CNS) not only during development, but also in adulthood, and become resident perivascular macrophages and microglia [1–3]. Data suggest that there is a continuous and lifelong turnover of perivascular brain macrophages replenished by bone marrow–derived cells. Furthermore, it has been demonstrated that bone marrow cells are recruited to sites of axonal degeneration [4,5] or to beta-amyloid depositions in Alzheimer disease animal models [6,7] to become functional microglia-like cells. The exact cellular subtype of myeloid precursors developing into microglia is not known, but parenchymal microglial cells have been reported to be phenotypically very similar to uncommitted myeloid precursors having “empty” major histocompatibility complex (MHC) class II, comparable cystein protease profiles, and the capacity to differentiate into dendritic-like cells [8,9]. Thus, bone marrow–derived myeloid cells (BM-MC) might reflect microglial precursors and may serve as a natural vehicle for CNS cell and gene therapy [2,10].

Efficient removal of apoptotic cells and cellular debris by macrophages during acute injury or degenerative diseases is essential for repair of the damaged tissue, inflammation resolution, and disease recovery [11–13]. Recently, we discovered in vitro that the microglial triggering receptor expressed on myeloid cells-2 (TREM2) stimulates phagocytosis and down-regulates inflammatory signals in microglia via the signaling adaptor molecule DAP12 [14]. Both clearance of tissue debris by phagocytosis and a protective cytokine milieu have been implicated as positive factors for tissue repair in multiple sclerosis (MS) [15]. Furthermore, the microglial cytokine milieu determines the outcome of the inflammatory process in the animal model of MS, experimental autoimmune encephalomyelitis (EAE) [16–18]. Therefore, clearance of cellular debris and resolution of inflammation are important for recovery and repair in neuroinflammatory diseases.

## Methods

### Isolation and Flow Cytometry Analysis of Microglia Derived from the CNS

Cortex, cerebellum, and spinal cord of normal or EAE-diseased mice were isolated and homogenized. Homogenates were filtrated through a 40-μM nylon mesh in a 50-ml tube, diluted with PBS and centrifuged (1,500 rpm) at room temperature for 5 min. Microglia and macrophages were separated through a density gradient. The cells were suspended in 70% Percoll (Amersham Biosciences, http://www.amershambiosciences.com) and overlaid with 37% and 30% gradient. The density gradient was centrifuged at 2,000 rpm for 30 min at room temperature. Myelin collected in the 30% Percoll layer was removed. The majority of microglia/macrophages were found in the interface of 37% and 70% Percoll. Cells were obtained from this interface and were washed from the Percoll with PBS. Cells were confirmed to be microglia/macrophages by flow cytometry analysis with specific biotinylated antibodies directed against CD11b (BD Biosciences Pharmingen, http://www.bdbiosciences.com) followed by streptavidin-PE (Dianova, http://www.dianova.de). For flow cytometry analysis, cells were incubated with a monoclonal rat anti-mouse TREM2 antibody (R&D Systems, http://www.rndsystems.com) and visualized with goat anti-rat FITC (Dianova).

### Isolation and Culture of BM-MC

Isolation of myeloid precursors was performed by a modified method published by Willenbring and collaborators [19]. In detail, bone marrow cells were isolated from adult 6–8-wk-old female C57BL/6 mice (Charles River, Sulzfeld, Germany) from the medullary cavities of the tibia and femur of the hind limbs. Removal of erythrocytes was performed by lysis with hypotonic solution. Cells were cultured in DMEM medium (Invitrogen, http://www.invitrogen.com) containing 10% fetal calf serum (Pan Biotech, http://www.pan-biotech.com) and 10 ng/ml of granulocyte macrophage–colony stimulating factor (GM-CSF) (R&D Systems) in 75-cm^2^ culture flasks (Greiner Bio-One, http://www.gbo.com). After 24 h, non-adherent cells were collected and re-seeded in fresh 75-cm^2^ culture flasks. Medium was changed after 5 d and BM-MC were collected for experiments after 10–11 d.

### Isolation and Culture of Monocytes

Peripheral blood was collected from adult C57BL/6 mice. Erythrocytes were depleted by hypotonic lysis buffer. Cells were plated on culture dishes in RPMI medium (Invitrogen) containing 10% fetal calf serum (Pan Biotech). Cells were cultured for several hours at 37 °C in 10% CO_2_. After trypsinization, adherent cells were collected and used for phagocytosis experiments.

### Lentiviral Vector System and Bone Marrow–Derived Myeloid Cell Transduction

Lentiviral vectors of the third generation, (pLenti6/V5, Invitrogen) were used for transduction of BM-MC. The blasticidin-resistance gene in the pLenti6/V5 vector was replaced by a second cytomegalovirus (CMV) promoter, and the green fluorescent protein (GFP) gene was subcloned downstream of the *TREM2* gene, which was obtained from primary murine microglia and placed under the original CMV promoter. The correct nature of all cloned sequences was confirmed by automated sequencing (Seqlab, Göttingen, Germany). Lentiviral transduction was performed as described [14]. Titers of concentrated viral particles ranged between 1 × 10^8^ and 1 × 10^9^ transducing units/ml. Lentiviral particles and 6 μg/ml polybrene (Sigma, http://www.sigmaaldrich.com) were added at day 2 to cultured BM-MC. Supernatant was removed 24 h after infection and replaced with DMEM medium containing 10% fetal calf serum with GM-CSF. In all experiments, the efficiency of BM-MC transduction was >90% as determined by the number of BM-MC expressing the GFP molecule and by immunohistochemistry.

### Flow Cytometry Analysis of BM-MC

BM-MC transduced with the TREM2 vector or the control GFP vector were first incubated for Fc-receptor blockade by CD16/CD32 antibody (BD Biosciences Pharmingen) and then stained with either biotin-conjugated anti-CD45, anti-CD11b, anti-CD11c, anti-CD86, anti-CD80, anti-I-A^b^ (MHC class II), anti-CD36, anti-Sca-1, anti-c-kit, anti-CD133 (BD Biosciences Pharmingen), anti-F4/80 (Serotec Laboratories, http://www.serotec.com), or anti-mouse CCR7 (Alexis, Lausen, Switzerland) followed by CyChrome-conjugated streptavidin (BD Biosciences Pharmingen) or biotin-conjugated anti-goat IgG and Cy5-conjugated streptavidin (Dianova). Furthermore, cells were stained with anti-TREM2 monoclonal antibodies (R&D Systems) followed by biotin-conjugated anti-rabbit IgG (Dianova) and CyChrome-conjugated streptavidin (BD Biosciences Pharmingen). Cells obtained from EAE spinal cord were stained with anti-CD45 antibody (BD Biosciences Pharmingen) and CyChrome-conjugated streptavidin (BD Biosciences Pharmingen), or with anti-TREM2 monoclonal antibody followed by biotin-conjugated anti-rabbit IgG and CyChrome-conjugated streptavidin. Isotype-matched control antibodies (Sigma) were used as negative control. Analysis was done with a FACScalibur flow cytometer (BD Biosciences Pharmingen). Live gating was performed with propidium iodide (Sigma).

### RT-PCR Analysis of *TREM2* and *DAP12* Gene Transcripts

RNA was isolated from BM-MC by RNeasy Mini Kit (Qiagen, Valencia, California, United States). Reverse transcription of RNA was performed with SuperScript III reverse transcriptase (Invitrogen) and hexamer random primers (Roche Molecular Biochemicals, Mannheim, Germany). For semi-quantification, all samples were normalized with GAPDH amplification.

### Western Blot Analysis of ERK

BM-MC were transduced with the TREM2 vector. Then 2 × 10^5^ cells were cultured on dishes, pre-coated with 10 μg/ml monoclonal antibody directed against TREM2 (R&D Systems) or with 10 μg/ml isotype control antibody (Sigma). After stimulation for 1 h, cells were lysed in reducing sample buffer for Western blot analysis. Phosphorylation of ERK and the total amount of ERK were determined by immunodetection with anti-phospho-ERK and anti-ERK antibodies, respectively (both from Cell Signaling Technology, Beverly, Massachusetts, United States).

### Microsphere Bead-Phagocytosis Assay

BM-MC were transduced with the TREM2 vector or the control GFP vector. Transduced BM-MC or cultured monocytes (5 × 10^4^) obtained from peripheral blood of C57BL/6 mice were cultured on dishes that had been pre-coated with 10 μg/ml monoclonal antibody directed against TREM2 (R&D Systems) or with 10 μg/ml isotype control antibody (Sigma). After 1 h, 100 μl of red fluorescent microsphere beads solution (Fluoresbrite Polychromatic Red Microspheres, 1.00 μm, Polysciences; http://www.polysciences.com) was added to 1 ml of cultured cells for 1 h. After a washing step, all cells from three independent fields (at 63× magnification) were counted under a fluorescence microscope, and phagocytosis of microsphere beads by BM-MC or cultured monocytes was analyzed. Relative change in phagocytosis was observed due to interexperimental variations. Data are shown as the relative change in phagocytosis between cells cultured on anti-TREM2 antibody and control antibody. For phagocytosis of apoptotic neural cells, the percentage of phagocytosis (the number of phagocytes having taken up at least two beads/the number of all BM-MC × 100) was calculated. After counting the number of cells under a fluorescence microscope, cells were collected and the percentage of GFP+ (green signal) plus bead+ (red signal) was quantified by flow cytometry. The threshold for defining the uptake of at least two beads was determined by the intensity of the flow cytometry signal. The relative change in phagocytosis was determined due to interexperimental variations. Data are shown as the relative change in phagocytosis between cells cultured on anti-TREM2 antibody and control antibody. In blocking experiments, ERK inhibitor PD98059 (20 μM) (Calbiochem, http://www.emdbiosciences.com/g.asp?f=CBC/home.html) was added 60 min before stimulation of TREM2.

### Phagocytosis Assay of Apoptotic Neurons

Neuron-enriched cells were derived from embryonic E18 mouse (C57BL/6) hippocampus and cultured for 5–10 d. Okadaic acid was added for 3 h at a concentration of 30 nM to induce apoptosis. Apoptotic cell membranes were labeled with CellTracker CM-DiI membrane dye (http://probes.invitrogen.com). After incubation, apoptotic cells were washed twice and added to the transduced myeloid precursor culture or cultured monocytes at an effector:target ratio of 1:20. One hour after the addition of apoptotic cells, the number of BM-MC or cultured monocytes having phagocytosed apoptotic cell membranes was counted under a confocal fluorescence microscope (Leica, Wetzlar, Germany). Apoptotic cells were counted in three different areas at 60× magnification. The amount of phagocytosis was also confirmed by flow cytometry. In blocking experiments, ERK inhibitor PD98059 (20 μM) (Calbiochem) was added 60 min before stimulation of TREM2. Moreover, 48 h after the addition of apoptotic neuron–enriched cultures, the cells were collected and used for semiquantitative real-time RT-PCR of inflammatory gene transcripts.

### Analysis of Inflammatory Gene Transcripts by Real-Time RT-PCR

RNA was isolated by the RNeasy Mini Kit (Qiagen) from 2 × 10^5^ TREM2 or GFP-transduced myeloid precursors after cross-linking by TREM2-specific antibodies for 1 h followed by addition of 100 ng/ml lipopolysaccharide (LPS) (Sigma) or coculturing with apoptotic cells. Furthermore, RNA was isolated from total spinal cord, peritoneal lymph nodes, and spleen of EAE-diseased mice 2 d after intravenous application of TREM2-transduced BM-MC, GFP-transduced BM-MC, or PBS control (6 d after first clinical signs of EAE). Reverse transcription of RNA was performed with SuperScript III reverse transcriptase (Invitrogen) and hexamer random primers (Roche Molecular Biochemicals). Quantitative RT-PCR with specific oligonucleotides was performed with Test SYBR Green PCR Master Mix (Qiagen) using the ABI 5700 Sequence Detection System (Perkin Elmer, http://www.amebioscience.com) and amplification protocol for the ABI 5700 Sequence Detection System. Amplification specificity was proven by a melting curve. Results were analyzed with the ABI 5700 Sequence Detection System v.1.3 after establishing the reaction efficiency for each primer pair. Quantification using the delta-CT method was carried out, and standard curves were used for each plate.

### ELISA Assay for Interleukin-10

Enzyme-linked immunosorbent assay (ELISA) for interleukin-10 (IL-10) was used to determine the levels of IL-10 released from myeloid cells after stimulation. TREM2 or GFP-transduced myeloid precursors were stimulated by cross-linking TREM2-specific antibodies for 1 h followed by addition of 100 ng/ml LPS (Sigma) or by coculturing with apoptotic cells. Supernatant was collected after 24 h, and the level of IL-10 was determined by ELISA according to the manufacturer's instructions (QuantikineM mouse IL-10, R&D Systems).

### Induction and Cell Therapy of EAE

Adult 7–9-wk-old female C57BL/6 mice (obtained from Charles River Laboratories, http://www.criver.com) were injected in the tail base bilaterally with 200 μl of an innoculum containing 100 μg of myelin oligodendrocyte glycoprotein peptide 35–55 (amino acids MEVGWYRSPFSRVVHLYRNGK; Seqlab) and 1 mg of Mycobacterium tuberculosis H37 Ra (Difco, http://www.bd.com) in incomplete Freund adjuvant (Difco). Pertussis toxin (200 ng; List Biological Laboratories, http://www.listlabs.com) was injected at day 0 and at day 2 after immunization. Clinical signs were scored as follows: 0, no clinical signs; 1, complete limp tail; 2, complete limp tail and abnormal gait; 3, one hind-limb paraparesis; 4, complete hindlimb paraparesis; 5, fore- and hind-limb paralysis or moribund. Only mice having disease onset (clinical score of 1 or more) at day 14 were used for experiments. TREM2-transduced (5 × 10^6^ cells in PBS), GFP-transduced BM-MC (5 × 10^6^ cells in PBS), or vehicle control (PBS) were injected intravenously under light anesthesia in EAE-diseased mice at the day of the first clinical symptoms, or at day 4 after first clinical symptoms.

### Immunohistochemistry

Animals were perfused transcardially with 0.125 M PBS followed by 4% paraformaldehyde. After post-fixation in fresh fixative, tissue was cryoprotected at 4 °C in 0.125 M PBS containing 2% DMSO and 10% glycerol. Tissue was embedded in OCT (Sakura, http://www.sakura.com), frozen on dry ice, and stored at −80 °C before cryosectioning on a Leica CM1900 cryostat at −20 °C. For Iba1 and CD45 staining, sections were refixed in 4% paraformaldehyde for 1 h and then immunostained with purified polyclonal rabbit antibody directed against Iba1 (Wako, http://www.wako-chem.co.jp/english/labchem/productinf.htm) and secondary-fluorescence Cy3-conjugated antibody directed against rabbit IgG (1:200, Dianova), or with monoclonal antibody directed against CD45 (1:100, BD Biosciences Pharmingen) and a secondary-fluorescence Cy3-conjugated antibody directed against rat IgG (1:200, Dianova). For lysosome-associated membrane protein 2 (Lamp2) staining, sections were refixed in methanol for 5 min and then immunostained by monoclonal rat antibody directed against Lamp2 (1:100, Abcam, Hiddenhausen, Germany) and a secondary-fluorescence Cy3-conjugated antibody directed against rat IgG (1:200). Fluorescence images were collected by confocal laser scanning microscopy with a 40× objective (Leica). For quantification of Lamp2-positive cells, random images from frozen sections were scanned by a blinded observer and analyzed by another blinded observer. Random images from frozen sections of different time points after cell injection were also used to count the numbers of GFP+ cells that had migrated into the spinal cord.

Histological examination of immune cell infiltrates, axonal injury, and demyelination was performed as described previously [20]. In brief, mice were perfused with 4% paraformaldehyde in PBS at indicated time points. After removal of the spinal cord and fixation in 4% buffered formalin, spinal cord was dissected and embedded in paraffin. After microwave pretreatment in 10 mM citric acid, the following antibodies were used for immunohistochemical analysis of the spinal cord tissue: monoclonal rat anti-mouse MAC3 (clone M3/84, dilution: 1:200, BD Biosciences Pharmingen) for macrophage staining, monoclonal rat anti-human/mouse CD3 (clone CD3–12, dilution: 1:200, Serotec Laboratories) for T cells, monoclonal mouse anti-amyloid precursor protein (APP) (clone 22C11, dilution: 1:100, Chemicon, Temecula, California, United States) for axonal degeneration, monoclonal mouse anti-SMI 32 (dilution: 1:200, Sternberger Monoclonals, Lutherville, Massachusetts, United States) for the detection of dephosphorylated neurofilament, and polyclonal rabbit anti-myelin basic protein (dMBP, AB5864, dilution 1:2000, Chemicon) for degraded MBP. The following secondary antibodies were utilized: for CD3 and MAC3, a biotinylated goat anti-rat immunoglobulin (RPN1005, dilution 1:200, Amersham Biosciences) and for APP, SMI32 sheep anti-mouse immunoglobulin (RPN1001, dilution 1:200, Amersham Biosciences). Immunohistochemistry was processed by using the avidin–biotin amplification bridge method with peroxidase as a substrate.

Inflammatory spinal cord lesions were defined in each animal on the basis of the MAC3 staining and were further analyzed by immunohistochemistry and luxol fast blue (LFB) staining. The number of MAC3+ cells, CD3+ cells, APP and dMBP-positive deposits, SMI-positive axons, and the level of demyelination in LFB staining were quantified on three lumbal spinal cord sections in at least three mice per group using an Olympus BX51 microscope (Olympus, Hamburg, Germany) equipped with a digital camera (SIS Color View) attached to a computer with software for image analysis (AnalySIS; Soft Imaging System, http://www.soft-imaging.net). Quantification of MAC3+ cells, CD3+ cells, APP- and dMBP-positive deposits was performed by a blinded observer.

In LFB staining, total white matter and demyelinated areas from three cross sections were measured by planimetry, and the area of demyelination was expressed as a percentage of the total area of the white matter. Decreased LFB staining represents either demyelination or a loss of myelin due to axonal destruction. For each animal, the mean percentage of demyelination of the individual sections was calculated.

In SMI32 staining, the relative number of dephosporylated axons in the spinal cord white matter was determined using an axon grid (Olympus) under ×1,000 magnification. Thus, the relative number of SMI32-positive axons covered by 25 crosses per grid was counted in at least seven eye fields per mouse (*n* = 3 mice per group).

### Statistical Analysis

Data were analyzed by SPSS software (Statistical Package for Social Sciences version 14.0 for Windows; http://calcnet.mth.cmich.edu/org/spss/index.htm). Analysis of variance (ANOVA) was applied to experiments with three or more experimental groups. Groups showing differences after testing with ANOVA were re-analyzed by pairwise *t*-test. The *p*-values presented throughout the Results are derived from the pairwise *t*-test, with the exception of the ANOVA results that gave a higher *p*-value. The sample sizes in animal experiments were preplanned taking into consideration the German animal protection law.

## Results

### Expression of TREM2 within the CNS

**Figure 1:**
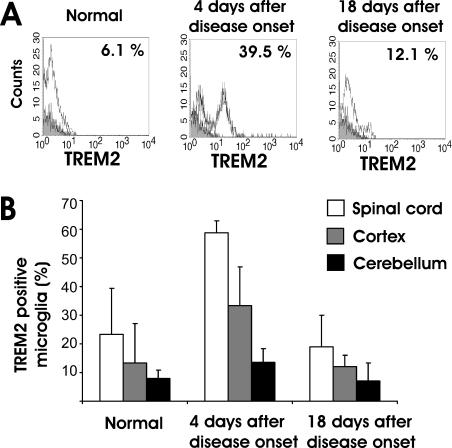
Expression Profile of TREM2 in Normal and Diseased CNS (A) Flow cytometry analysis of TREM2 (open tracings) expression on CD11b+ cells derived from the cortex of normal and EAE-diseased mice at day 4 after onset of clinical symptoms as well as from EAE-diseased mice at day 18 after onset of clinical symptoms. Isotype controls are shown in grey-filled tracings. TREM2 expression is detected on CD11b+ microglia/macrophages in the cortex of EAE-diseased mice. The percentages of TREM2+ cells are indicated in the upper-right corner of each histogram. Selected representative histograms are shown. (B) Quantitative analysis of TREM2 expression on microglia by flow cytometry gated for CD11b+ cells. TREM2 expression is detected only on a minority of microglia and perivascular macrophages in the normal CNS tissue, but is up-regulated and detected in the spinal cord on the majority of CD11b+ cells at day 4 after onset of clinical symptoms of EAE. Data are presented as mean ± SEM. Tissues were derived from four EAE and four normal mice.

TREM2 has been shown to be expressed on certain subtypes of myeloid cells, particularly osteoclasts, immature dendritic cells, and microglia [21]. To study the expression kinetics of TREM2 during EAE, we performed flow cytometry analysis of TREM2 on freshly isolated microglia and resident CNS cells identified by immmunostaining for CD11b. Flow cytometry analysis demonstrated TREM2 expression on a minority of microglial cells under normal conditions in adult C57BL/6 mice (Figure 1A). Next, we induced EAE by immunization with the myelin oligodendrocyte glycoprotein peptide 35–55. At day 4 after onset of clinical symptoms of EAE, TREM2 was detected on approximately half of the CD11b+ cells in the spinal cord, the tissue region mostly affected by the inflammatory lesions (Figure 1A and 1B). In detail, the percentage of TREM2 and CD11b+ cells in the spinal cord increased from 23.3% (standard error of the mean [SEM] 16.1) in the normal tissue to 58.8% (SEM 4.2) at day 4 after onset of clinical signs of the disease (Figure 1B). The up-regulation of TREM2 was detected mainly in the spinal cord, but not in the cortex and cerebellum, indicating that TREM2 expression is associated with the inflammatory lesions. The up-regulation of TREM2 on CD11b+ cells was not long lasting and returned back to almost normal levels at day 18 after onset of clinical EAE symptoms (Figure 1B).

### Lentiviral Transduction of Bone Marrow–Derived Myeloid Precursor Cells with TREM2

**Figure 2:**
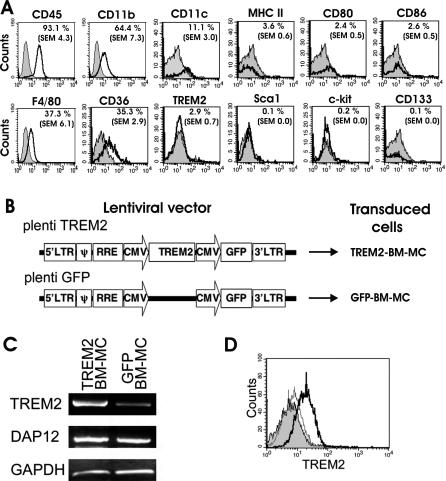
Characterization and TREM2 Transduction of BM-MC (A) Flow cytometry analyses of BM-MC. Bone marrow cells were cultured for 10 d in GM-CSF–containing medium and then analyzed by flow cytometry with specific antibodies (open tracings) directed against CD45, CD11b, CD11c, MHC class II, CD80, CD86, F4/80, CD36, TREM2, Sca1, c-kit, and CD133. Cells showed expression of CD45, F4/80, CD11b, CD11c, and CD36. The percentage of positive cells is indicated in the upper-right corner of each histogram. Isotype controls are shown in grey-filled tracings. (B) Lentiviral vector design. To express TREM2 in BM-MC, the mouse TREM2 coding sequence was cloned under the CMV promoter followed by a second CMV promoter and GFP (plenti TREM2). The same vector without TREM2 was used as a control (plenti GFP). (C) Gene transcripts in myeloid cells transduced with TREM2 vector (TREM2-BM-MC) or control GFP vector (GFP-BM-MC) for TREM2, DAP12, and the housekeeping gene GAPDH were analyzed by RT-PCR. Gene transcripts for TREM2 were strongly detected in TREM2-BM-MC and weakly in GFP-BM-MC. Both TREM2-transduced myeloid cells and control GFP vector–transduced myeloid cells showed gene transcription for DAP12, the TREM2 adapter and signaling molecule. (D) Flow cytometry analysis of TREM2-transduced BM-MC (bold line) and GFP-transduced BM-MC (narrow line), both labeled with TREM2-specific antibodies. Isotype control antibody is shown in filled grey. Cell surface expression of TREM2 was detected on myeloid cells after lentiviral transduction with TREM2, but not on control GFP vector–transduced myeloid cells.

To study the involvement of microglial TREM2 in EAE, we isolated bone marrow–derived myeloid precursors, lentivirally transduced them with TREM2, and applied them as putative precursors of microglia in diseased mice. Bone marrow cells were isolated from female adult C57BL/6 mice and cultured in GM-CSF–containing medium for 10–11 d. Under these culture conditions, the number of CD11b+ cells increased at least 20-fold as determined by counting the CD11b+ cells before and after culture. Flow cytometry analysis of the cultured BM-MC showed expression of CD45, CD11b, CD11c, CD36, and F4/80 (Figure 2A). No expression of MHC class II, CD80, CD86, CD133, Sca1, and c-kit (CD117) was detected, indicating that BM-MC still have an immature phenotype, but without expressing stem cell surface markers (Figure 2A). Importantly, no cell surface expression of the microglial receptor TREM2 was detected (Figure 2), and very low levels of *TREM2* gene transcripts were observed (Figure 2). However, RT-PCR demonstrated gene transcripts of the TREM2-associated signaling molecule DAP12 (Figure 2). To study the role of the microglial receptor TREM2 on myeloid cells in EAE, we transduced BM-MC with a lentiviral vector expressing both TREM2 and the marker GFP under two distinct CMV promoters or a corresponding control GFP vector (Figure 2B). Transduction efficiency of BM-MC by vesicular stomatitis virus-G protein–pseudotyped lentiviral vectors was regularly greater than 90% (Table S1). The genetically modified myeloid precursor cells exhibited gene transcripts and protein expression of TREM2 as demonstrated by RT-PCR and flow cytometry analysis (Figure 2C and 2D).

### Increased Phagocytosis and Anti-Inflammatory Cytokine Signaling by TREM2-Transduced Myeloid Precursors In Vitro

**Figure 3:**
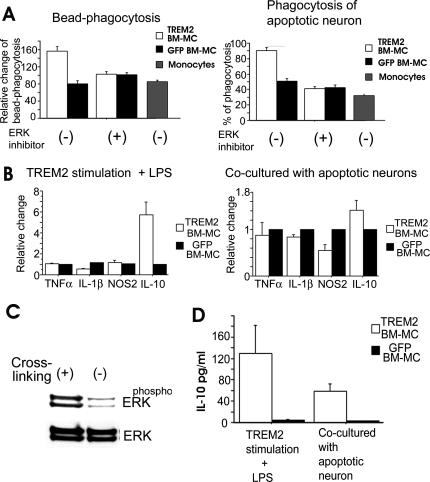
Myeloid Cells Transduced with TREM2 Showed Increased Phagocytic and Anti-Inflammatory Activity In Vitro (A) TREM2-transduced myeloid cells showed increased phagocytosis of beads and of apoptotic neuron–enriched cultures. Analysis of the phagocytosis of beads (left graph), TREM2-transduced myeloid cells (open bars), control GFP vector–transduced myeloid cells (filled bars), or cultured monocytes (grey bars) after stimulation with TREM2-specific antibodies either untreated (−) or treated with ERK inhibitor (+). Phagocytosis assay (right graph) of apoptotic cells derived from neuron-enriched cultures by TREM2-transduced myeloid cells (open bars), control GFP vector–transduced myeloid cells (filled bars), or cultured monocytes (grey bars), either treated with ERK inhibitor (+) or untreated (−). Transduction of myeloid cells (BM-MC) with TREM2 increased the phagocytosis of beads and apoptotic cells that were dependent on ERK phosphorylation. Data are presented as mean ± SEM. Unpaired *t*-test: *p* < 0.001 (TREM2 versus GFP) for BM-MC without ERK inhibitor and with bead phagocytosis, and *p* < 0.001 (TREM2 versus GFP) for BM-MC without ERK inhibitor and with phagocytosis of apoptotic neurons. Five independent experiments were performed, and each of them was done in quadruplicate. (B) Anti-inflammatory activity of TREM2 stimulation. Myeloid cells were transduced with TREM2 (open bars) or control GFP vector (filled bars). Myeloid cells were cultured on plates coated with TREM2 cross-linking antibodies (left graph). One hour later, cells were stimulated with LPS. Gene transcript levels for TNFα, IL-1β, NOS2, and IL-10 were quantified by real-time PCR after 48 h. TREM2-transduced myeloid cells showed reduced gene transcript levels of IL-1β and NOS2 and an increased level of IL-10. In addition, apoptotic cells derived from neuron-enriched cultures were added to myeloid cells (right graph). Gene transcript levels for TNFα, IL-1β, NOS2, and IL-10 were quantified by real-time PCR after 48 h. Data are presented as mean ± SEM. Unpaired *t*-test: *p* < 0.001 (TREM2 versus GFP) with TREM2 plus LPS for IL-1, *p* = 0.0205 (TREM2 versus GFP) with TREM2 plus LPS for IL-10, *p* = 0.0149 (TREM2 versus GFP) with coculture for IL-1, and *p* = 0.012 (TREM2 versus GFP) with coculture for NOS2. Four independent experiments were performed. (C) Phosphorylation of ERK after stimulation of TREM2. Myeloid cells were transduced with TREM2 and cultured for 1 h on plates coated with TREM2 cross-linking antibodies (+) or isotype control antibodies (−). Cross-linking of TREM2 increased the amount of phosphorylated ERK (phospho ERK) in relation to total ERK (ERK). (D) TREM2-transduced myeloid cells showed an increased release of IL-10 after stimulation of TREM2 plus LPS or coculture with apoptotic neuron–enriched cultures. Myeloid cells were transduced with TREM2 (open bars) or with control GFP vector (filled bars), cultured on plates coated with TREM2 cross-linking antibodies, and stimulated with LPS 1 h later or supplemented with apoptotic cells derived from neuron-enriched cultures. The amount of IL-10 released in the supernatant was determined by ELISA. Data are presented as mean ± SEM. Unpaired *t*-test: *p* = 0.044 (TREM2 versus GFP) for TREM2 plus LPS, and *p* = 0.0054 (TREM2 versus GFP) for coculture. Four independent experiments were performed.

Stimulation of TREM2 has been shown to induce phagocytosis in primary microglia [14]. In order to determine the effects of TREM2 expression on phagocytic activity of BM-MC, we analyzed phagocytosis of microsphere beads and apoptotic cells by TREM2-transduced BM-MC. Phagocytosis of microsphere beads was increased in TREM2-stimulated TREM2-transduced BM-MC compared to control BM-MC and cultured monocytes (Figure 3A). Likewise, phagocytosis of apoptotic membrane fragments derived from mixed neuron-enriched cultures was increased after gene transduction of TREM2. In detail, 93.3% (SEM 6.6) of BM-MC phagocytosed apoptotic neurons after transduction with TREM2, while only 50.5% (SEM 5.6) of BM-MC phagocytosed apoptotic neurons after transduction with the control GFP vector (Figure 3A). Increased phagocytosis of beads and apoptotic cells after TREM2 stimulation was completely neutralized by the ERK inhibitor PD98059, suggesting that ERK signaling was involved in TREM2-mediated phagocytosis by BM-MC (Figure 3A). TREM2 cross-linking antibodies stimulated phosphorylation of ERK in TREM2-transduced BM-MC (Figure 3), but did not modify cell surface expression of MHC class II, CD80, CD86, CD36, CCR7, and CD11c (Figure S1).

Next, we asked whether phagocytosis is associated with down-regulation of inflammatory cytokine gene transcription. Stimulation of TREM2-transduced BM-MC by solely TREM2-specific antibody did not modulate gene transcription of inflammatory mediators (Figure S1). Therefore, we studied the effect of TREM2 stimulation on LPS-treated BM-MC. Quantitative real-time RT-PCR of several cytokines was performed 48 h after TREM2 cross-linking of TREM2-transduced BM-MC and control GFP vector–transduced BM-MC, which have been stimulated with 100 ng/ml LPS. Interestingly, stimulation by TREM2 antibody 1 h before LPS treatment showed increased IL-10 (5.7-fold [1.3 SEM]) and decreased interleukin-1β (IL-1β; 0.5-fold [0.1 SEM]) gene transcription in TREM2-transduced BM-MC compared to control GFP vector–transduced BM-MC (Figure 3B). Moreover, we investigated gene transcript levels of cytokines in BM-MC cocultured for 48 h with apoptotic mixed neuron-enriched cultures. Gene transcripts of IL-1β and nitric oxide synthase-2 (NOS2) were decreased in TREM2-transduced BM-MC after challenge with apoptotic cells (Figure 3B). Transduction by TREM2 enabled BM-MC to release a substantial amount of IL-10 after stimulation as determined by ELISA (Figure 3D). In detail, TREM2-transduced BM-MC released 130 pg/ml IL-10 (SEM 52) after stimulation with LPS- and TREM2-specific antibodies (Figure 3D).

### Selective Migration of TREM2-Transduced Myeloid Cells to Inflammatory Lesions

**Figure 4:**
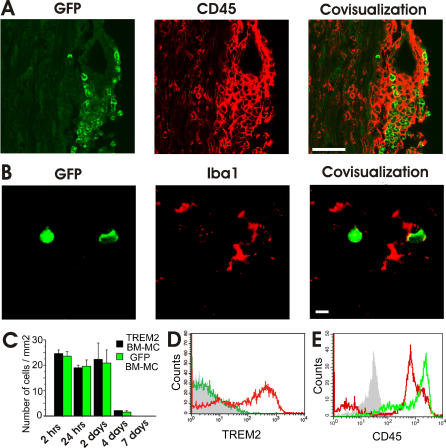
Migration of TREM2-Transduced Myeloid Cells into EAE Lesions (A) Detection of invaded myeloid cells in the spinal cord of EAE-diseased mice. Histological sections were obtained at day 2 after intravenous injection of GFP-transduced cells in EAE-diseased mice. Myeloid cells were injected at day 4 after first clinical signs of EAE. Sections were labeled with CD45-specific antibodies. Injected myeloid cells expressed CD45 and accumulated in inflammatory lesions. Scale bar indicates 50 μm. (B) No Iba1 antigen was detected on invaded myeloid cells by double immunofluorescence labeling. GFP+ cells were analyzed in the spinal cord of EAE-diseased mice 4 d after intravenous injection of GFP-transduced myeloid cells. Myeloid cells were applied 4 d after first clinical signs of the disease. Invaded cells showed round appearance, reminiscent of activated macrophage/microglial cells. Scale bar indicates 10 μm. (C) Quantification of cells invaded into the spinal cord of EAE-diseased mice. TREM2-transduced BM-MC or GFP-transduced BM-MC were injected intravenously at day 4 after the first clinical signs of the disease appeared. The BM-MCs migrated into spinal cord lesions within 2 h after intravenous application and remained there for 2–4 d. No GFP+ cells were detected within the spinal cord at day 7 after intravenous application. Data are presented as mean ± SEM. (D) Flow cytometry analysis of TREM2 expression on TREM2-transduced and GFP-transduced myeloid cells obtained from the spinal cord of EAE-diseased mice at day 2 after application. Expression of TREM2 was detected on myeloid cells transduced with TREM2 (red tracing), but not on cells transduced with GFP (green tracing). Tracings shown are from cells gated for GFP. Control antibody isotype is shown as grey-filled tracing. (E) Flow cytometry analysis of CD45 expression on GFP-transduced myeloid cells before injection (green tracing) and on GFP+ cells invaded into the spinal cord (red tracing) at day 4 after injection. Engrafted myeloid cells showed reduced levels of CD45 expression, reminiscent of microglia. Tracings shown are from cells gated for GFP. Control antibody isotype is shown as grey-filled tracing.

We also analyzed migration of TREM2 or control GFP vector–transduced BM-MC into the CNS after application in mice intravenously. In healthy mice, no GFP+ cells were detected on the microscopic level in the CNS tissue after injection of 5 × 10^6^ BM-MC. Likewise, we failed to detect GFP-transduced BM-MC in the CNS of mice afflicted by EAE, when 5 × 10^6^ cells were injected intravenously at day 1 after appearance of the first clinical symptoms. Then, we injected 5 × 10^6^ TREM2 or control GFP vector–transduced BM-MC intravenously into EAE-diseased mice at 4 d after onset of first clinical symptoms, at a time period reflecting the peak of the disease. The GFP+ cells were detected in the spinal cord of EAE-diseased mice, preferentially in perivascular inflammatory lesions (Figure 4A). In detail, analysis of inflammatory perivascular EAE lesions at day 2 after cell therapy with TREM2-BM-MC demonstrated a density of 261 cells/mm^2^ of invaded GFP+ cells (standard deviation [SD] 82). The majority (86%) of invaded TREM2-BM-MC was located within the perivascular area with a distance of less than 50 μm from the vessel wall. A substantial proportion (14%) of the invaded GFP+ cells was also detected within the spinal cord parenchyma.

Invaded myeloid cells were round in shape and were clearly positive for the common hematopoietic surface marker CD45 (Figure 4A), but not for the microglial typical marker Iba1 (Figure 4B). Myeloid cells applied intravenously migrated into the spinal cord lesions within 2 h (Figure 4C), indicating that they did not home in to other organs before entering the nervous tissue. No difference in the amount of invaded cells in EAE lesions was detected when comparing TREM2-transduced and control GFP vector–transduced BM-MC (Figure 4C). The cells were detected in the spinal cord for 2–3 d after administration. Finally, we did not detect substantial numbers of GFP+ cells in the spinal cord at day 4 after injection (Figure 4C). At day 7, no GFP+ cells were detected in the spinal cord, indicating that they either underwent apoptosis or migrated away from the spinal cord. Next, flow cytometry analysis of GFP+ cells isolated from the spinal cord was performed. Myeloid cells transduced with TREM2 showed expression of TREM2 within the spinal cord tissue, while no TREM2 expression was detected on GFP-transduced cells (Figure 4D). At day 4 after injection of BM-MC in EAE-diseased mice, 40.0% (SEM 7.2) of GFP+ cells showed reduced CD45 antigen expression compared to BM-MC before intravenous application (Figure 4E). Thus, BM-MC migrated into the spinal cord independently of TREM2 expression and showed down-regulated CD45 expression, but did not acquire a mature microglial phenotype and failed to show long-lasting engraftment.

### Tissue Debris Clearance and Creation of an Anti-Inflammatory Cytokine Milieu by TREM2-Transduced Myeloid Cells

**Figure 5:**
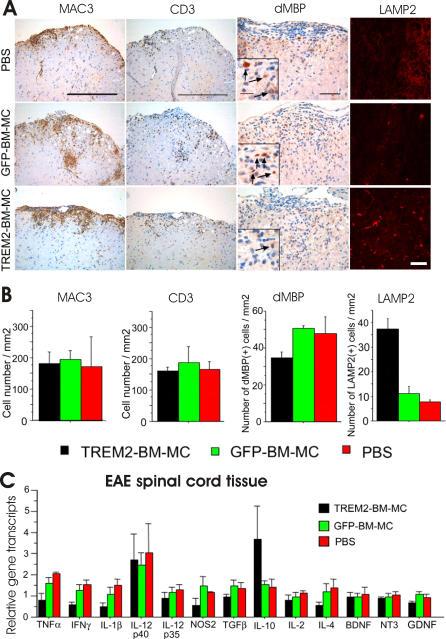
Increased Phagocytic Activity and Removal of Degenerated Myelin in the Spinal Cord by TREM2-Transduced Myeloid Cells (A) Immunostaining of macrophages (MAC3), T cells (CD3), degenerated MBP (dMBP), and Lamp2 in the spinal cord of EAE-diseased mice at day 2 after injection of TREM2-transduced myeloid cells (TREM-BM-MC), GFP-transduced myeloid cells (GFP-BM-MC), or vehicle control (PBS). Cells were injected intravenously in EAE-diseased mice at day 4 after first clinical signs of the disease. Scale bars for MAC3 and CD3 indicate 300 μm. Scale bars for dMBP and Lamp2 indicate 50 μm. (B) Quantification of the number of MAC3- and CD3+ cells, degenerative MBP, and LAMP2-positive cells. EAE-diseased mice were injected at day 4 after first clinical signs of the disease with TREM2-transduced myeloid cells (TREM2-BM-MC), GFP-transduced myeloid cells (GFP-BM-MC), or vehicle control (PBS). At day 2 after cell injection, spinal cords were removed, stained, and analyzed. Macrophages were labeled by MAC3 and T cells by CD3-specific antibodies. Cell therapy by myeloid precursors did not affect the spinal cord immune cell infiltrates. Furthermore, quantification of cells stained for degenerated MBP (dMBP) in the spinal cord of EAE-diseased mice at day 2 after injection was performed, and the amount of degenerated MBP was reduced by cell therapy with TREM2-transduced myeloid cells. Quantification of Lamp2-positive cells (LAMP2) in spinal cord lesions of EAE-diseased mice at day 2 after cell injection was performed. The number of Lamp2-positive cells showing phagocytic activity was increased by the intravenous injection of TREM2-BM-MC. For quantitative analysis, three lumbar spinal cord sections of three mice per group were examined. Data are presented as mean ± SEM. ANOVA, followed by unpaired *t*-test: *p* = 0.0422 (TREM2 versus GFP) for dMBP, and *p* = 0.0069 (TREM2 versus GFP) for LAMP2. (C) Anti-inflammatory cytokine milieu in the spinal cord after treatment with TREM2-transduced myeloid cells. Real-time RT-PCR of cytokines and growth factors at day 6 after first clinical signs of the disease and 2 d after injection of TREM2-transduced myeloid cells (*n* = 3), control GFP vector–transduced myeloid cells (*n* = 3), or PBS as vehicle control (*n* = 3). Relative gene transcript levels of TNFα, IFNγ, and IL-1β were decreased. ANOVA followed by unpaired *t*-test: *p* = 0.0324 (TREM2 versus GFP) for TNFα, *p* = 0.0111 (TREM2 versus PBS) for TNFα, *p* = 0.0068 (TREM2 versus PBS) for IFNγ, and *p* = 0.0215 (TREM2 versus PBS) for IL-1β. Each PCR experiment was performed in quadruplicate.

Since TREM2-transduced BM-MC showed increased phagocytosis of apoptotic cells and suppressed pro-inflammatory gene transcription in vitro, we tested their in vivo potential in clearance of tissue debris in EAE. In total, 5 × 10^6^ myeloid cells transduced either with TREM2 or with the control GFP vector, or with PBS alone, were injected intravenously in EAE-diseased mice at day 4 after disease onset. No difference in the number of infiltrating T cells (CD3) and macrophages (MAC3) into the nervous tissue was detected at day 2 after application of TREM2-transduced cells, GFP-transduced cells, or PBS control (Figure 5A and 5B; Table 1). To determine phagocytosis in situ, we analyzed the lysosomal activity within the inflammatory spinal cord lesions by immunostaining of methanol-fixed tissue with antibody directed against Lamp2. An increased number of cells showing Lamp2 staining was detected in the spinal cord of EAE-diseased mice at day 2 after treatment with TREM2-transduced myeloid cells (37.5 [SEM 4.2] cells/mm^2^) compared to mice treated with GFP-transduced myeloid cells (11.0 [SEM 3.1] cells/mm^2^) or vehicle control (7.7 [SEM 0.9] cells/mm^2^) (Figure 5A and 5B). These data indicated that TREM2-transduced myeloid cells show increased lysosomal degradation associated with phagocytosis of debris.

**Table 1:**
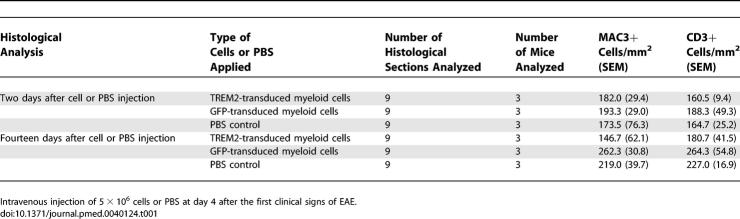
Histological Quantification of CNS Immune Cell Invasion

Next, we investigated in vivo the distribution of degenerated myelin basic protein (MBP) immunoreactivity, which has been phagocytosed and degraded in lysosomes by myeloid cells in inflammatory lesions. Degenerated MBP was detected with a specific antibody directed against an epitope unmasked during the process of myelin degeneration [22]. The number of cells immunostained for degenerated MBP in the spinal cord of EAE-diseased mice was reduced at day 2 after treatment with TREM2-transduced myeloid cells (34.5 [SEM 3.5] cells/mm^2^) compared to mice treated with GFP-transduced myeloid cells (50.5 [SEM 1.5] cells/mm^2^) or vehicle control (48.0 [SEM 9.0] cells/mm^2^) (Figure 5A and 5B).

Finally, we analyzed whether increased phagocytosis by TREM2-transduced myeloid cells is also associated with an anti-inflammatory cytokine milieu in the spinal cord lesions. Quantitative RT-PCR analysis at day 2 after cell injection demonstrated a significant decrease of gene transcription of tumor necrosis factor-α (TNFα), interferon-γ (IFNγ), and IL-1β, and an increase of gene transcript of IL-10 (3.7-fold) in the spinal cord of the TREM2-BM-MC–injected EAE-diseased mice (Figure 5C). Creation of an anti-inflammatory cytokine milieu was solely detected within the spinal cord, but not in secondary lymphoid organs such as the spleen and lymph nodes (Figure S2), indicating that TREM2-transduced myeloid cells specifically acted within the CNS. Furthermore, a change in the spinal cord cytokine profile was later followed by a slightly decreased number of infiltrating immune cells. In detail, at day 14 after treatment, 180.7 (SEM 41.5) CD3+ cells/mm^2^ and 146.7 (SEM 62.1) MAC3+ cells/mm^2^ were counted in the spinal cord tissue of TREM2-BM-MC–treated animals, while 264.3 (SEM 54.8) CD3+ cells/mm^2^ and 262.3 (SEM 30.8) MAC3+ cells/mm^2^ were observed in GFP-BM-MC–treated mice (Table 1).

### Disease Amelioration and Reduced Tissue Damage by TREM2-Transduced Myeloid Precursor Cells

**Figure 6:**
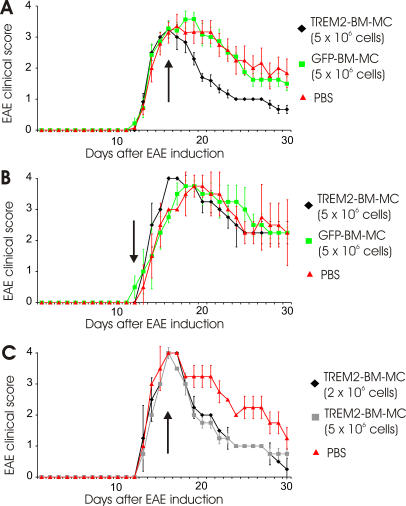
TREM2-Transduced Myeloid Cells Ameliorated EAE (A) Treatment of EAE-diseased mice by TREM2-transduced myeloid cells at the peak of the disease. Clinical EAE score of mice injected at day 4 after first clinical signs of the disease (arrow) with TREM2-transduced myeloid cells (black tracing), control GFP vector–transduced myeloid cells (green tracing), or PBS (red tracing). Intravenous application of 5 × 10^6^ TREM2-transduced myeloid cells ameliorated the clinical score of EAE. Data are presented as mean ± SD. Mice per group: *n* = 6 or *n* = 7. ANOVA followed by unpaired *t*-test: *p* = 0.0015 (TREM2 versus GFP) and *p* = 0.0063 (TREM2 versus PBS) at day 20; *p* = 0.0045 (TREM2 versus GFP) and *p* = 0.0046 (TREM2 versus PBS) at day 30. (B) TREM2-transduced myeloid cells injected at the onset of the disease do not affect EAE. Clinical EAE score of mice injected 1 d after the first clinical signs of the disease (arrow) with 5 × 10^6^ TREM2-transduced myeloid cells (black), 5 × 10^6^ control GFP vector–transduced myeloid cells (green), or PBS (red). No effect on the clinical EAE score was observed. Data are presented as mean ± SD. Mice per group: *n* = 3. (C) Treatment of EAE by TREM2-transduced myeloid cells. Clinical EAE score of mice injected at day 4 after first clinical signs of the disease with 2 × 10^6^ TREM2-transduced myeloid cells (black), 5 × 10^6^ TREM2-transduced myeloid cells (grey), or PBS control (red). Intravenous application of 2 × 10^6^ or 5 × 10^6^ TREM2-transduced myeloid cells ameliorated the clinical score of EAE. Data are presented as mean ± SD. Mice per group: *n* = 3. ANOVA followed by unpaired *t*-test: *p* = 0.0023 (2 × 10^6^ cells versus PBS) and *p* = 0.0068 (5 × 10^6^ cells versus PBS) at day 20; *p* = 0.0107 (2 × 10^6^ cells versus PBS) at day 30.

TREM2-transduced BM-MC showed an effect on the EAE disease course, leading to early and almost complete recovery from clinical symptoms. In total, 5 × 10^6^ BM-MC were injected intravenously at day 4 after onset of first clinical symptoms of EAE, which coincides with the disease peak with ongoing tissue destruction. Already 2 days after cell injection, mice improved in clinical performance (Figure 6A). At 7 days, the mean clinical score was reduced from 2.5 (SD 0.4) after GFP-transduced BM-MC application to 1.3 (SD 0.1) after TREM2-transduced BM-MC application (Figure 6A; Table S2). While no difference was detectable in the cumulative clinical score before and on the day of cell injection, the cumulative clinical score was reduced from 33.8 (SD 2.5) after injection of 5 × 10^6^ GFP-transduced BM-MC to 17.6 (SD 6.0) after injection of 5 × 10^6^ TREM2-transduced BM-MC (Table S2). Importantly, no changes in either the disease course or in cumulative clinical scores were observed in PBS-injected mice and control GFP vector–transduced BM-MC–injected animals (Table S2). TREM2-transduced BM-MC were only effective in ameliorating the disease symptoms if injected at day 4 after onset of clinical symptoms. TREM2-transduced cells injected at day 1 after clinical onset of EAE did not interfere with the disease course, indicating that TREM2-tranduced BM-MC do not prevent the initiation phase of EAE, but act during the recovery and repair phase of the disease (Figure 6B). Finally, we analyzed the effect of different cell amounts of TREM2-transduced BM-MC. A reduced cell amount of 2 × 10^6^ TREM2-transduced BM-MC was as effective in ameliorating the clinical disease course as 5 × 10^6^ TREM2-transduced BM-MC (Figure 6C).

**Figure 7:**
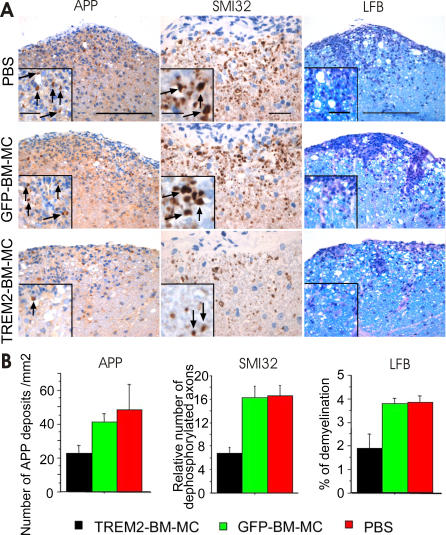
Application of TREM2-Transduced Myeloid Cells Reduced Axonal and Myelin Injury (A) Reduced spinal cord axonal injury and decreased demyelination after cell therapy by TREM2-transduced myeloid cells. Axonal injury and damage were analyzed by immunostaining with specific antibodies directed against APP and dephosphorylated neurofilament (SMI32) at day 14 after cell application. Demyelination was determined by LFB staining at day 14 after cell application. Mice were injected at day 4 after first clinical signs of EAE by TREM2-transduced myeloid precursor cells (TREM2-BM-MC), GFP-transduced BM-MC (GFP-BM-MC), or PBS control. Representative histological pictures are shown. Scale bars for APP and LFB indicate 200 μm, and for inserts indicate 50 μm. Scale bar for SMI32 indicates 50 μm, and for insert indicates 20 μm. (B) Quantification of axonal damage and demyelination 14 d after cell application in EAE with TREM2-transduced myeloid cells (black bars), control GFP vector–transduced myeloid cells (green bars), or vehicle PBS control (red bars). Level of axonal injury (APP-positive deposits/mm^2^), axonal damage (relative number of SMI32-positive axons), and demyelination (loss of LFB, as a percentage) were significantly reduced after cell therapy with TREM2-transduced BM-MC. For quantitative analysis, at least three lumbar spinal cord sections of three mice per group were investigated. Data are presented as mean ± SEM. ANOVA followed by unpaired *t*-test: *p* = 0.0425 (TREM2 versus GFP) and *p* = 0.0759 (TREM2 versus PBS) in APP; *p* = 0.0012 (TREM2 versus GFP) and *p* = 0.0098 (TREM2 versus PBS) in SMI32; *p* = 0.0465 (TREM2 versus GFP) and *p* = 0.00450 (TREM2 versus PBS) in LFB.

Apart from its clinical effect, TREM2-transduced cells protected and supported repair of the spinal cord tissue. At day 14 after cell therapy with TREM2-transduced myeloid cells, a clear reduction of axonal damage and demyelination was detected as determined by immunostaining for APP, dephosphorylated neurofilament (SMI32), and staining of myelin (LFB) (Figure 7A). In detail, mice treated with 5 × 10^6^ TREM2-transduced BM-MC showed, at day 14 after cell injection, fewer axonal APP-stained deposits per mm^2^ (23.0 [SEM 4.6]) compared to either 5 × 10^6^ GFP-transduced (41.6 [SEM 5.0]) or PBS-injected EAE-diseased mice (45.3 [SEM 16.0]) (Figure 7B). Likewise, the relative number of dephosphorylated axons was reduced to 6.8 (SEM 0.9) compared to either GFP-transduced (16.3 [SEM 1.6]) or PBS-injected EAE animals (16.5 [SEM 1.6]) (Figure 6B). In addition, TREM2-BM-MC–treated mice revealed a reduced percentage of demyelination (1.8% [SEM 0.6]) compared to GFP-BM-MC–treated mice (3.8% [SEM 0.2]) or PBS-treated mice (3.9% [SEM 0.3]) (Figure 7B). Thus, TREM2-transduced BM-MC–treated mice showed a clear protective and repair-promoting effect on spinal cord tissue in EAE.

## Discussion

In this study, we have shown that intravenously applied TREM2-transduced myeloid precursors ameliorate clinical symptoms and induce recovery from EAE. The TREM2-transduced myeloid cells migrated into the CNS lesions, induced myelin debris clearance, and created an anti-inflammatory cytokine milieu. The treated mice showed less axonal injury and reduced demyelination. The data demonstrate that TREM2+ myeloid cells are involved in tissue debris clearance and resolution of inflammation and suggest a novel approach to the therapy of neuroinflammatory disease.

TREM2 on microglia fulfills an important function for CNS tissue homeostasis. Loss-of-function mutations of TREM2 in humans result in inflammatory neurodegeneration, loss of myelin, basal ganglia calcification, and bone abnormalities [23–25]. TREM2 is selectively expressed on immature dendritic cells, osteoclasts, and microglia, but not on monocytes [21,26]. TREM2 in osteoclasts is involved in resorption of bones, also requiring phagocytic activity [24,27]. In microglial cells, TREM2 promotes phagocytosis and anti-inflammatory cytokine signaling [14]. Recently, it has been shown that TREM2 is expressed on macrophages infiltrating CNS tissue from the circulation, and that TREM2 acts to inhibit cytokine production by macrophages in response to several toll-like receptor ligands [28].

In this study, we have demonstrated that the gene transfer of TREM2 in bone marrow myeloid precursor cells supports phagocytosis of beads and apoptotic neuronal cells and, in addition, shows strong anti-inflammatory activity. Both effects are required to promote the repair of injured CNS tissue by engulfing tissue debris and shutting off inflammatory processes. We injected the TREM2-transduced myeloid cells into the circulation of EAE-diseased mice and detected these cells in their spinal cord lesions as well as in the spleen, but failed to detect any change in the cytokine profile in the spleen and lymph nodes. Possibly, the ligand of TREM2, which appears to be a component of apoptotic neuronal cell membranes [14], is not present in normal spleen and lymph nodes, but is located in the spinal cord tissue that has the lesion. The TREM2-transduced myeloid cells migrated into the spinal cord lesions within 2 h following intravenous application, showed increased lysosomal activity, cleared the myelin debris, and created an anti-inflammatory cytokine milieu within the spinal cord. The invaded myeloid cells remained within the lesions for several days, but were not detected after 1 wk, indicating that they either evaded the lesions or underwent apoptosis or autophagy.

Our data show that TREM2 on myeloid cells supports an essential function of the CNS tissue phagocytes. The phagocytic function of macrophages and microglia has been implicated in the removal of myelin debris during EAE [29]. Particularly, macrophages appear to fulfill an important role in the clearance of tissue debris, since depletion of macrophages impairs CNS remyelination [30,31]. Rapid clearance of myelin debris is required since components of the myelin such as Nogo prevent axonal regeneration and axonal remodeling [32]. Axonal remodeling has been shown to contribute to recovery and repair in EAE [33]. Therefore, the reduced amount of demyelination and axonal damage after therapy with TREM2-transduced myeloid precursor cells might also be partially explained by improved remyelination and axonal remodeling via facilitated engulfment of myelin debris. Alternatively, reduced demyelination and less axonal injury after therapy with TREM2-transduced myeloid precursor cells could be a positive effect of the improved resolution of inflammation and the anti-inflammatory cytokine milieu, both limiting the inflammation-induced tissue damage.

Furthermore, TREM2 of myeloid cells created an anti-inflammatory cytokine milieu in the spinal cord of EAE-diseased mice. Particularly, the gene transcript level of TNFα was reduced, while that of IL-10 increased after application of TREM2-transduced myeloid precursor cells. Increased gene transcripts of IL-10 might be directly due to invaded TREM2-transduced myeloid precursors stimulated within the lesion sites, but we cannot exclude the possibility that TREM2-transduced myeloid precursors act indirectly via a secondary immune organ to stimulate a regulatory immune cell type migrating into the CNS. IL-10 has profound regulatory effects on the immune system and is vital for the recovery phase of EAE. Interestingly, IL-10 gene transcripts are increased in the CNS during the recovery phase of EAE [34]. IL-10 is involved in the disease recovery and resolution of inflammation because remission is impaired in IL-10–deficient mice [35]. Furthermore, the involvement of CD4+ CD25+ regulatory T cells in the recovery from EAE can be narrowed to their production of IL-10 [36]. Most importantly, exogenous administration of IL-10 is only truly effective at ameliorating EAE when targeted directly to the CNS [37]. Thus, IL-10 produced by the TREM2-transduced myeloid precursor cells during engulfment of myelin debris might favor recovery from EAE.

The TREM2-transduced myeloid cells were applied during ongoing EAE at a time point when the antigen-priming inflammatory phase has been completed and the recovery phase started. No interference with immune cell infiltration was observed at day 2 after cell injection since EAE-diseased mice treated with TREM2-transduced myeloid cells showed the same amount of infiltrating T cells and macrophages as EAE animals injected with control GFP vector–transduced myeloid cells. However, at day 14 after cell injection, EAE-diseased mice treated with TREM2-transduced myeloid cells demonstrated a slightly reduced number of MAC3+ macrophages and CD3+ T cells within the inflammatory lesions, indicating that the cell therapy by TREM2-transduced myeloid cells promoted resolution of inflammatory cells. TREM2-transduced myeloid cells did not improve clinical symptoms of EAE, if applied at day 1 of clinical symptoms. We failed to detect the GFP-labeled cells within the CNS after application of cells at this early time point, indicating that migration of cells into the lesion side is required for therapeutic efficacy.

EAE has been studied in DAP12-deficient mice [38], which might have impaired TREM2 signaling. While TREM2 of myeloid precursor cells improved ongoing disease by possibly acting locally within the CNS, DAP12-deficient animals failed to develop EAE due to impaired antigen priming during the inflammatory phase [38]. Furthermore, myeloid precursor cells used in our study failed to express MHC class II and costimulatory molecules required for antigen presentation to auto-reactive CD4+ T cells. In this respect, myeloid precursor cells were very similar to resting microglial cells, which lack MHC class II and CD86 expression under normal conditions [16].

In EAE, auto-reactive CD4+ TH1 cells secrete pro-inflammatory cytokines such as IFNγ and TNFα initiating a CNS inflammatory reaction, which is then amplified within the CNS by microglia [16,39,40]. The inflammatory phase is either promptly resolved by shutting off inflammation or deteriorates in a neurodegenerative phase, which involves axonal and neuronal injury. This secondary progressive neurodegenerative phase, particularly with axonal tissue loss, appears to be resistant to anti-inflammatory therapies and predicts the clinical outcome in MS patients [41,42]. Most experimental approaches in EAE aim at skewing the cytokine profile of myelin-specific TH cells from TH1 to TH2 by altered peptide ligands [43,44], hydroxymethyl-glutaryl coenzyme A reductase inhibitors (“statins”) [45], or DNA vaccination combined with gene delivery of interleukin-4 (IL-4) [46]. Neuroprotective and repair-promoting strategies of EAE are relatively unexplored, most probably due to the limited capacity of protein therapeutics to pass the blood–brain barrier and enter the target tissue. Targeting of cells into the lesioned CNS offers the possibility of a site-directed cellular effect and local release of therapeutic proteins. Genetically modified myelin-specific CD4+ TH1 cells over-expressing the neurotrophic factor, nerve growth factor, have been applied intravenously to treat EAE [47], but T cells were auto-reactive in nature and nerve growth factor also acted directly on immune cells such as monocytes.

Recently, brain-derived neurospheres have been injected intravenously and have been successfully used for cell therapy of EAE inducing repair and immunomodulatory activity [48,49], but these adult neural precursors are not a suitable cellular source for broad clinical applications. In contrast, bone marrow is an accessible source of myeloid precursor cells and, in addition, these cells can also be expanded to sufficient cell numbers from the peripheral blood of patients following bone marrow mobilization. To create a microglia-like phenotype, we transduced myeloid cells with the TREM2 receptor and treated EAE by intravenous cell injection.

Thus, we conclude that TREM2-transduced myeloid precursor cells applied intravenously ameliorate EAE either locally inside the CNS or indirectly via another regulatory immune cell type by clearance of tissue debris and resolution of inflammation, thereby opening new avenues for cell therapy of inflammatory and degenerative CNS diseases.

## Supporting Information

Figure S1 TREM2-Transduced Myeloid Precursor Cells Lack Up-Regulation of Immunoreceptors or Cytokines after Solely TREM2 Stimulation (A) No change in cell surface immunoreceptor expression of BM-MC after TREM2 stimulation. TREM2- or GFP-transduced BM-MC were cultured on plates coated with a cross-linking antibody specific for TREM2 or isotype control antibody. Cells were analyzed after 2 d of stimulation by flow cytometry. Stimulation of TREM2 did not change cell surface expression of CD80, CD86, MHC class II, CD11c, CCR7, and CD36. Grey filled line, negative control; blue line, TREM2-BM-MC with TREM2 stimulation; red line, TREM2-BM-MC without stimulation; green line, GFP-BM-MC with TREM2 stimulation; and yellow line, GFP-BM-MC without stimulation.
(B) No change in cytokine gene transcription of BM-MC after solely TREM2 stimulation. TREM2- or GFP-transduced BM-MC were cultured on plates coated with a cross-linking antibody specific for TREM2 or isotype control antibody. Cells were analyzed after 24 h of stimulation by real-time RT-PCR of TNFα, IL-1β, NOS2, and IL-10 to determine gene transcription of TREM2-transduced (open bars) and control GFP vector–transduced (filled bars) myeloid cells after cross-linking by TREM2-specific antibodies. No change in gene transcript levels of inflammatory mediators was detected after stimulation of TREM2 solely. Data are presented as mean ± SEM, *n* = 4.
(1.7 MB PDF)

Figure S2 Therapy of EAE by TREM2-Transduced Myeloid Cells Did Not Change the Cytokine Profile within the Spleen and Lymph Nodes The cytokine gene transcript levels in the spleen (A) and lymph nodes (B) were unchanged after injection of TREM2-transduced cells into EAE-diseased mice. Real-time RT-PCR of pro-inflammatory cytokines was performed at day 6 after first clinical signs of the disease and 2 d after injection of TREM2-transduced myeloid cells (*n* = 3), control GFP vector–transduced myeloid cells (*n* = 3), or PBS as vehicle control (*n* = 3). Gene transcript levels of TNFα, IFNγ, IL-1β, IL-4, IL-10, and NOS2 were unchanged. Data are presented as mean ± SEM.
(3.6 MB PDF)

Table S1 Transduction Efficiency of BM-MC by Lentiviral Vectors (24 KB DOC)

Table S2 Disease Scores of EAE (39 KB PDF)

Table S3 Oligonucleotide Sequences Used in This Study (29 KB PDF)

## Abbreviations

ANOVA: analysis of variance
APP: amyloid precursor protein
BM-MC: bone marrow–derived myeloid cell
CMV: cytomegalovirus
CNS: central nervous system
EAE: experimental autoimmune encephalomyelitis
ELISA: enzyme-linked immunosorbent assay
GFP: green fluorescent protein
GM-CSF: granulocyte macrophage–colony stimulating factor
IFNγ: interferon-γ
IL-10: interleukin-10
IL-1β: interleukin-1β
IL-4: interleukin-4
Lamp2: lysosome-associated membrane protein 2
LFB: luxol fast blue
LPS: lipopolysaccharide
MBP: myelin basic protein
MHC: major histocompatibility complex
MS: multiple sclerosis
NOS2: nitric oxide synthase-2
SD: standard deviation
SEM: standard error of the mean
TNFα: tumor necrosis factor-α
TREM2: triggering receptor expressed on myeloid cells 2
