# Supplementary material for: TREM2-Transduced Myeloid Precursors Mediate Nervous Tissue Debris Clearance and Facilitate Recovery in an Animal Model of Multiple Sclerosis
Source: PLoS Med. 2007 Apr 10;4(4):e124. doi: 10.1371/journal.pmed.0040124 (PMC1851623; doi:10.1371/journal.pmed.0040124)
Supplement: Supplementary file 1 [file pmed.0040124.sg001.pdf]

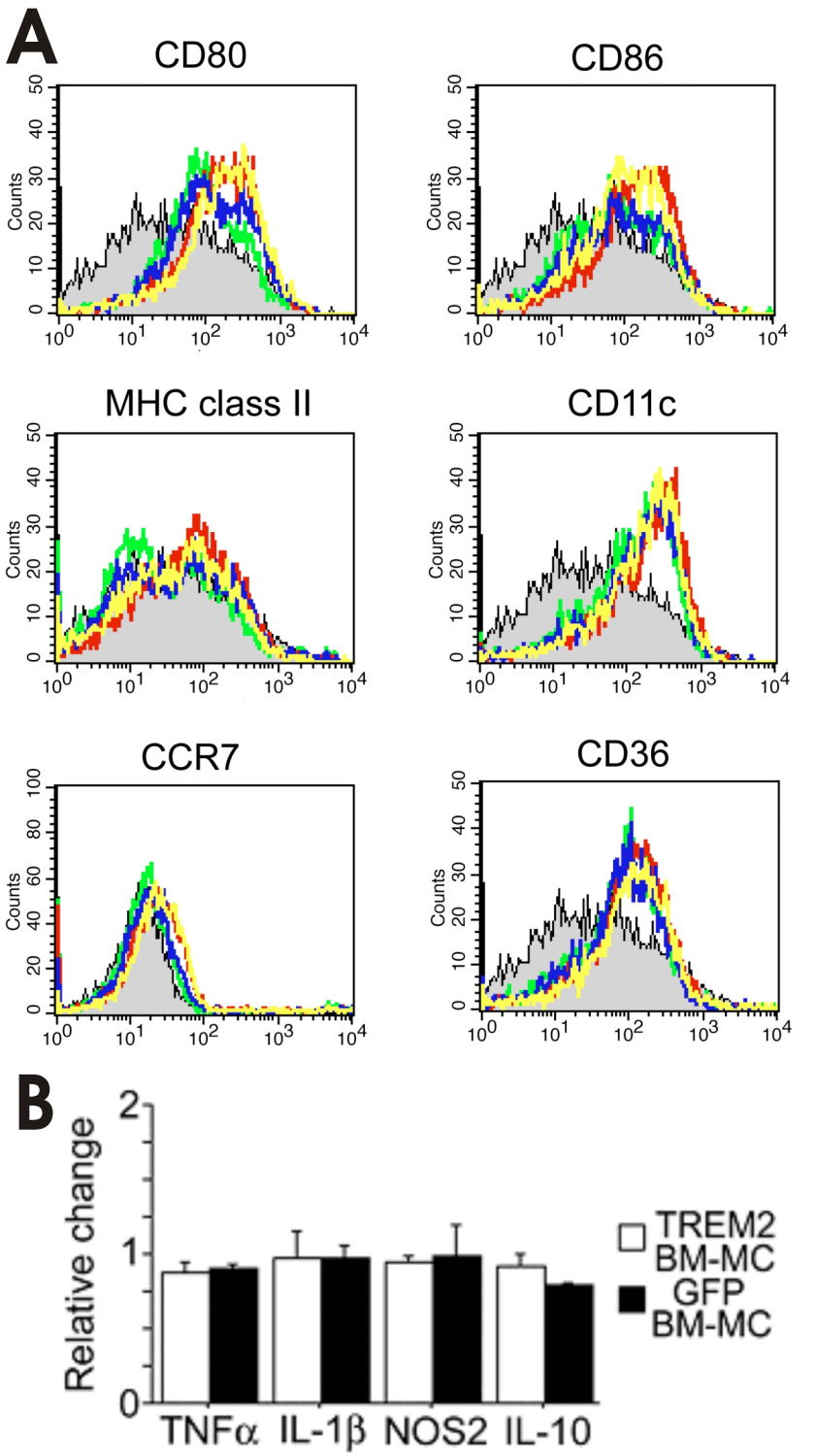
A
CD80
CD86
MHC class II
CD11c
CCR7
CD36
Counts
10^0 10^1 10^2 10^3 10^4
B
Relative change
TREM2 BM-MC
GFP BM-MC
TNFα IL-1β NOS2 IL-10
