# Supplementary material for: TREM2-Transduced Myeloid Precursors Mediate Nervous Tissue Debris Clearance and Facilitate Recovery in an Animal Model of Multiple Sclerosis
Source: PLoS Med. 2007 Apr 10;4(4):e124. doi: 10.1371/journal.pmed.0040124 (PMC1851623; doi:10.1371/journal.pmed.0040124)
Supplement: Supplementary file 2 [file pmed.0040124.sg002.pdf]

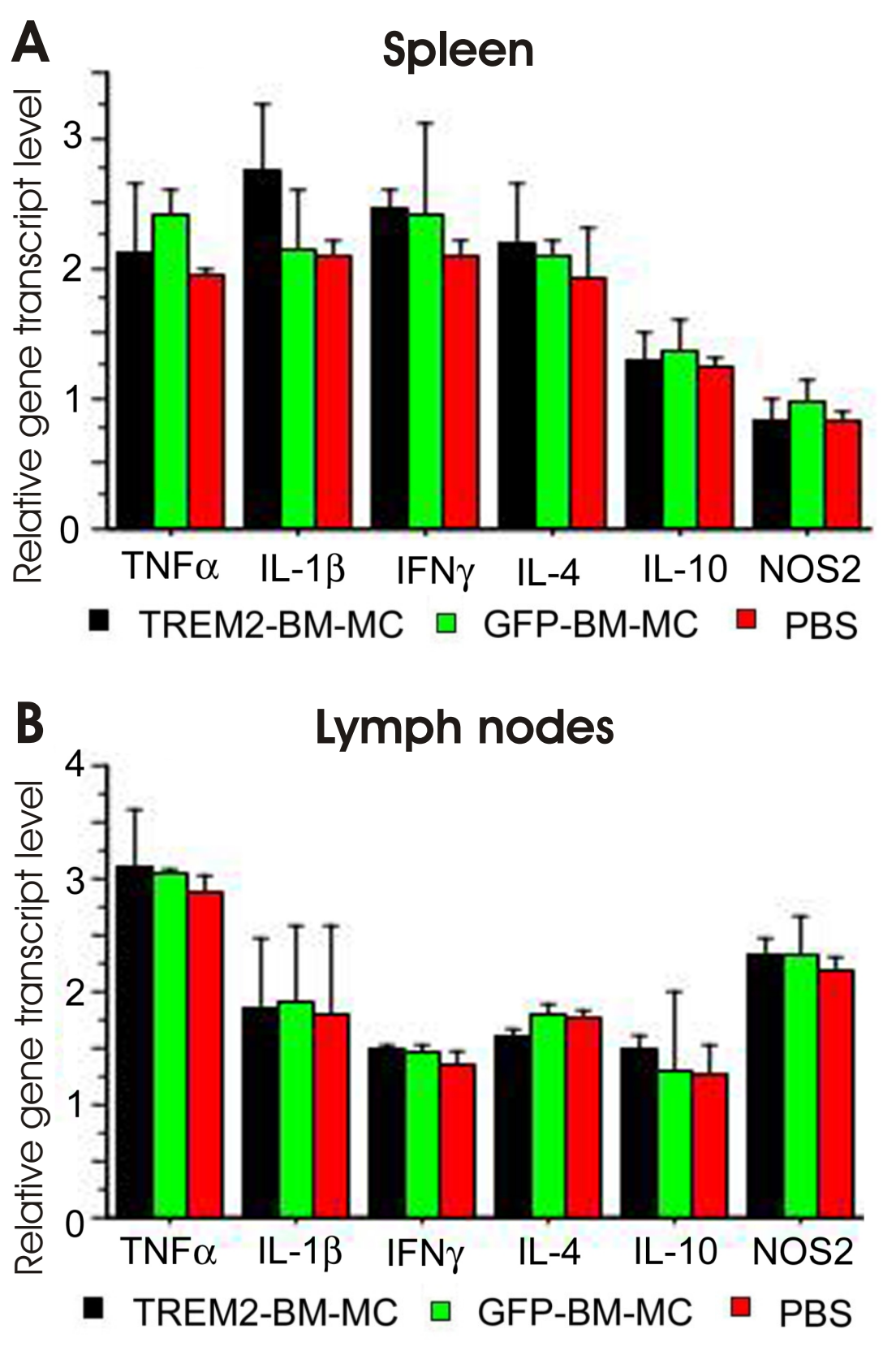
A
Spleen
Relative gene transcript level
0
1
2
3
TNFα
IL-1β
IFNγ
IL-4
IL-10
NOS2
TREM2-BM-MC
GFP-BM-MC
PBS
B
Lymph nodes
Relative gene transcript level
0
1
2
3
4
TNFα
IL-1β
IFNγ
IL-4
IL-10
NOS2
TREM2-BM-MC
GFP-BM-MC
PBS
