# Supplementary material for: TREM2-Transduced Myeloid Precursors Mediate Nervous Tissue Debris Clearance and Facilitate Recovery in an Animal Model of Multiple Sclerosis
Source: PLoS Med. 2007 Apr 10;4(4):e124. doi: 10.1371/journal.pmed.0040124 (PMC1851623; doi:10.1371/journal.pmed.0040124)
Supplement: Supplementary file 3 [file pmed.0040124.st001.doc]

**Transduction efficiency of BM-MC by lentiviral vectors**

| Vector type applied to BM-MC | Number of independent experiments | Percentage of cells showing expression of GFP |
| --- | --- | --- |
| plenti TREM2 vector expressing TREM2 and GFP | 6 | 93.8 % (SEM 1.8) |
| plenti GFP vector expressing GFP | 6 | 96.7 % (SEM 0.9) |
