# Supplementary material for: TREM2-Transduced Myeloid Precursors Mediate Nervous Tissue Debris Clearance and Facilitate Recovery in an Animal Model of Multiple Sclerosis
Source: PLoS Med. 2007 Apr 10;4(4):e124. doi: 10.1371/journal.pmed.0040124 (PMC1851623; doi:10.1371/journal.pmed.0040124)
Supplement: Supplementary file 4 [file pmed.0040124.st002.doc]

**Disease scores of EAE**

| Injection i.v. of 5x 10^6^ cells or PBS at day 4 after the first clinical signs of EAE | | | | |
| --- | --- | --- | --- | --- |
| Type of cells or PBS applied | Number of mice per group | Cumulative disease score (from day 1 to the day of cell injection) | Disease score on day of cell injection | Cumulative disease score (from the day of cell injection to day 30) |
| TREM2-transduced myeloid cells | 6 | 9.3 (SD 1.3) | 3.4 (SD 0.3) | 17.6 (SD 6.0) |
| GFP-transduced myeloid cells | 7 | 9.5 (SD 2.0) | 3.3 (SD 0.3) | 33.8 (SD 2.5) |
|   PBS control | 7 | 9.3 (SD 1.7) | 3.4 (SD 0.4) | 36.2 (SD 1.3) |
| Injection i.v. of 5x 10^6^ cells or PBS at day 1 after the first clinical signs of EAE | | | | |
| Type of cells or PBS applied | Number of mice per group | Cumulative disease score (from day 1 to the day of cell injection) | Disease score on day of cell injection | Cumulative disease score (from the day of cell injection to day 30) |
| TREM2-transduced myeloid cells | 3 | 10.5 (SD 0.5) | 4.0 (SD 0.0) | 50.3 (SD 1.8) |
| GFP-transduced myeloid cells | 3 | 10.5 (SD 1.5) | 3.8 (SD 0.3) | 50.5 (SD 3.0) |
|   PBS control | 3 | 9.0 (SD 0.0) | 3.8 (SD 0.3) | 48.7 (SD 2.8) |
| Injection i.v. of 2x 10^6^ cells, 5x 10^6^ cells or PBS at day 4 after the first clinical signs of EAE | | | | |
| Type of cells or PBS applied | Number of mice per group | Cumulative disease score (from day 1 to the day of cell injection) | Disease score on day of cell injection | Cumulative disease score (from the day of cell injection to day 30) |
| TREM2-transduced 2x 10^6^ cells | 3 | 10.8 (SD 0.7) | 4.0 (SD 0.0) | 32.3 (SD 0.8) |
| TREM2-transduced 5x 10^6^ cells | 3 | 9.8 (SD 0.25) | 4.0 (SD 0.0) | 30.5 (SD 0.5) |
|   PBS control | 3 | 11.1 (SD 0.4) | 4.0 (SD 0.0) | 47.3 (SD 1.0) |
