# Supplementary material for: TREM2-Transduced Myeloid Precursors Mediate Nervous Tissue Debris Clearance and Facilitate Recovery in an Animal Model of Multiple Sclerosis
Source: PLoS Med. 2007 Apr 10;4(4):e124. doi: 10.1371/journal.pmed.0040124 (PMC1851623; doi:10.1371/journal.pmed.0040124)
Supplement: Supplementary file 5 [file pmed.0040124.st003.doc]

**Oligonucleotide sequences used in this study**

Gene name	Accession no.	Oligonucleotide sequences for RT-PCR

GAPDH	NM_008084	forward: 5’–AACTTTGGCATTGTGGAAGG–3’

reverse: 5’–ACACATTGGGGGTAGGAACA–3’

TREM2	NM_011662	forward: 5’–ATGGGACCTCTCCACCAGTT–3’

reverse: 5’–TCACGTACCTCCGGGTCCA–3’

DAP12	NM_011662	forward: 5’–ATGGGGGCTCTGGAGCCCT–3’

reverse: 5’–TCATCTGTAATATTGCCTCTGTGT

Gene name	Accession no. Oligonucleotide sequences for real-time RT-PCR

GAPDH	NM_008084	forward: 5’–CTCCACTCACGGCAAATTCAA–3’

reverse: 5’–GATGACAAGCTTCCCATTCTCG–3’

TNFα	NM_013693	forward: 5’–CCGTCAGCCGATTTGCTATCT–3’

reverse: 5’–ACGGCAGAGAGGAGGTTGACTT–3’

IL-1β	NM_008361	forward: 5’–ACAACAAAAAAGCCTCGTGCTG–3’

reverse: 5’–CCATTGAGGTGGAGAGCTTTCA–3’

NOS2	NM_010927	forward: 5’–GGCAAACCCAAGGTCTACGTTC–3’

reverse: 5’–TACCTCATTGGCCAGCTGCTT–3’

TGFβ1	NM_011577	forward: 5’–AGGACCTGGGTTGGAAGTGG–3’

reverse: 5’–AGTTGGCATGGTAGCCCTTG–3’

IL-10	NM_010548	forward: 5’–AGGCGCTGTCATCGATTTCTC–3’

reverse: 5’–TGCTCCACTGCCTTGCTCTTA–3’

IFNγ	NM_008337	forward: 5’–TGCATCTTGGCTTTGCAGC–3’

reverse: 5’–GGCAGTAACAGCCAGAAACAGC–3’

IL-12p40	NM_008352	forward: 5’–TCACACTGGACCAAAGGGACT–3’

reverse: 5’–CCTCCTGGCAGGACACTGAA–3’

IL-12p35	NM_008351	forward: 5’–AAATGAAGCTCTGCATCCTGC–3’

reverse: 5’–TCACCCTGTTGATGGTCACG–3’

IL-2	NM_008366	forward: 5’–GTCAACAGCGCACCCACTT–3’

reverse: 5’–TGCTTCCGCTGTAGAGCTTG–3’

IL-4	NM_021283	forward: 5’–CACGGATGCGACAAAAATCA–3’

reverse: 5’–CTCGTTCAAAATGCCGATGA–3’

BDNF	NM_007540	forward: 5’–AGGCACTGGAACTCGCAATG–3’

reverse: 5’–AAGGGCCCGAACATACGATT–3’

NT3	NM_008742	forward: 5’–TCCTCAGCCATTGACATTCG–3’

reverse: 5’–GATCTCCCCCAGCACTGTGA–3’

GDNF	NM_010275	forward: 5’–GGGTGCGTTTTAACTGCCAT–3’

reverse: 5’–GCCCAAACCCAAGTCAGTGA–3’
